# Reproductive technologies needed for the generation of precise gene-edited pigs in the pathways from laboratory to farm

**DOI:** 10.5713/ab.22.0389

**Published:** 2022-11-14

**Authors:** Ching-Fu Tu, Shu-Hui Peng, Chin-kai Chuang, Chi-Hong Wong, Tien-Shuh Yang

**Affiliations:** 1 Division of Animal Technology, Animal Technology Research Center, Agricultural Technology Research Institute, Hsinchu 30093, Taiwan; 2 Department of Biotechnology and Animal Science, National Ilan University, Yilan 260007, Taiwan

**Keywords:** Farm, Gene-editing, New Breeding Technique, Pigs, Reproductive Biotechnology, Sustainability

## Abstract

Gene editing (GE) offers a new breeding technique (NBT) of sustainable value to animal agriculture. There are 3 GE working sites covering 5 feasible pathways to generate GE pigs along with the crucial intervals of GE/genotyping, microinjection/electroporation, induced pluripotent stem cells, somatic cell nuclear transfer, cryopreservation, and nonsurgical embryo transfer. The extension of NBT in the new era of pig breeding depends on the synergistic effect of GE and reproductive biotechnologies; the outcome relies not only on scientific due diligence and operational excellence but also on the feasibility of application on farms to improve sustainability.

## INTRODUCTION

Pig breeding has moved forward from the judging performance index to include genetic marker-assisted evaluation. With progress in understanding genomics and the implementation of advanced technologies, scientists can specifically alter a pig’s DNA to create novel changes or erase deleterious effects while disturbing animal wellbeing very little. Currently, gene-editing (GE) technology, especially the clustered regularly interspaced short palindromic repeat-CRISPR associated protein 9 (CRISPR/Cas9), has become the dominant approach, because it is characterized by a low technological barrier, high efficiency, and low cost. The generation of GE pigs has been successful with respect to improving meat quantity/quality, thermogenesis of the newborn, and disease resistance in the application of new breeding biotechnology (NBT), and these achievements have been recently reviewed [1].

Despite the simplicity of GE in the laboratory, the subsequent practice of CRISPR/Cas9 to generate GE pigs remains complex and ineffective, requiring enormous infrastructure and fiscal inputs. The pathway from laboratory DNA manipulation to on-farm neonatal delivery is a technically challenging production system and requires effective support from a series of biotechnologies. This review emphasizes the application of sound methods and improved outcomes while reducing the impacts on the generation of GE pigs.

## THE PATHWAYS OF GENERATION OF GENE-EDITED PIGS FROM LABORATORY TO FARM

**Figure 1:**
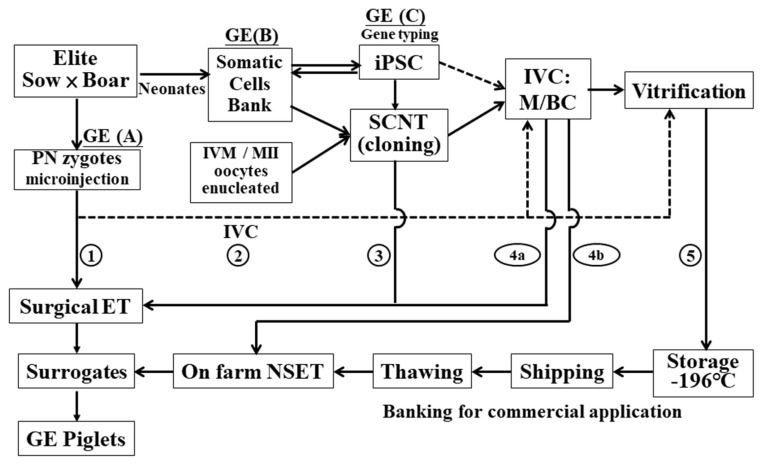
The pathways from laboratory to farm and the assisted biotechnology needed to generate and extend gene-edited (GE) pigs. (A), (B), and (C) are the sites able to conduct GE. Routes 1 to 5 represent different approaches to achieve successful outcomes with different requirements or paces. The solid and broken lines indicate the main and alternate courses, respectively. BCs, blastocysts; ET, embryo transfer; iPSC, induced pluripotent stem cells; IVC, *in vitro* culture; IVM, *in vitro* maturation; MII, metaphase II oocytes; M, morula; NSET, nonsurgical ET; PN, pronuclear.

A stepwise diagram (Figure 1) summarizes five different approaches together with the assisted biotechnologies required to generate GE pigs. The first begins with the direct microinjection of the GE DNA vectors into the pronucleus or the GE guide RNA (gRNA) and Cas9 mRNA into the cytoplasm of newly fertilized eggs (zygotes) that are surgically transferred into the fallopian tube of the surrogate estrus-synchronized to induce pregnancy. Allowing injected embryos (zygotes) to develop into morulas or blastocysts by *in vitro* culture (IVC) for embryo transfer (ET) or leading to cryopreservation/embryo banking for on-farm nonsurgical ET (NSET) is termed Way 2. However, in support of embryonic development, the current IVC system provides little substantial value, and misconduct always exists. Ways 3 to 5 start with GE somatic cell preparation followed by somatic cell nuclear transfer (SCNT) to produce reconstructed embryos. Way 3 performs surgical ET the day after SCNT. Way 4 includes viability selection of reconstructed embryos, IVC, and then either surgical ET (4a) or NSET (4b) to surrogates reared on farms. The feasible approach, or Way 5, is to nurture the reconstructed embryos, and those that develop into blastocysts shall be collected and vitrified for future on-farm extension/trading uses by adopting NEST at a later convenient time.

The crucial intervals of GE (I), induced pluripotent stem cell (iPSC) (II), SCNT (III), cryopreservation (IV), and NSET (V) are discussed in the present paper to realize the application of NBT in the GE pig production system from laboratory to farm.

### Gene editing

There are three methods available for GE, namely, zinc finger nuclease (ZFN), transcription activator-like effector nuclease (TALEN), and CRISPR/Cas9, and their application in pigs, particularly CRISPR/Cas9, has been recently reviewed [2,3]. The GE vectors or tools create genomic DNA double strains breakage (DSB) that requires immediate repair via nonhomologous end joining (NHEJ) [4] and/or microhomology-mediated end joining [5] to sustain cell survival with the risks of functional loss of gene editing. However, during chromosome cleavage and quick NHEJ, insertion and deletion (indel) may occur differently, resulting in various genetic conformations [6]. Although little chance of off-targeted editing has been claimed, there are several Cas nuclease modifications that attempt to enhance the precision of GE genetics [6]. A practical approach would be to use the two nearby gDNA/gRNAs for GE; thus, DSBs occur simultaneously to create a short fragment DNA deletion to simplify the genotype screening of GE cells/animals by polymerase chain reaction (PCR) without intense sequencing.

GE can be undertaken at three sites (A, B, and C in Figure 1). Site A uses newly fertilized eggs and microinjects porcine U6 promotor/gDNA and βactin (or others) promotor/Cas9 plasmid vectors directly into the pronucleus or microinjects into the cytoplasm with *in vitro* transcript gRNA and Cas9 mRNA (or protein). The porcine U6 promotor is a type III RNA polymerase III promoter that controls short hairpin RNA expression and effectively expresses gDNA to gRNA [7]. Site B is fibroblast GE, followed by SCNT with or without genotyping. Site C is a method that simultaneously includes GE, iPSC induction and candidate iPSC genotyping. At sites B and C, the cells will be reconstructed with enucleated oocytes, which mature *in vitro* (IVM) to the metaphase II (MII) stage. In the site A method, since GE is performed with no genotyping, mosaic founders carrying multiple genotypes with variant indels would usually be obtained. In site B, GE fibroblasts may generate cloned animals carrying deviant genotypes if not preselected; thus, prescreening is essential before SCNT to ensure that GE animals are purposely wanted. Site C allows the iPSC subclones to be carefully prescreened by PCR followed by amplicon sequencing to ensure that the subclones obtained are genotype correct. However, the efficacy of generating cloned pigs by using iPSCs and SCNT remains to be improved.

### Microinjection and electroporation

During maturation, mammalian oocytes enrich their protein factors for DNA replication, repair and transcription and make small RNAs and mRNA [8], as well as growth factors [9], to prepare for new fertilized egg cleavage and early-stage development before zygotic genome DNA expression turns on. All these proteins and nucleic acids enable the direct microinjection of the GE DNA vectors or gRNAs and Cas9 mRNA (or protein) to engage their functions in the host genome edited to generate GE pigs. We generated alpha1,3 galactosyltransferase (*GGTA1*) KO pigs by pronuclear microinjection with two gDNAs and Cas9 plasmid vectors [10] and CMP-N-acetylneuraminic acid hydroxylase (*CMAH*) KO pigs [11] and CD163 KO or exon 7 deleted pigs [12] by cytoplasm microinjection with two gRNAs and Cas9 mRNA. As the newly fertilized eggs replicate DNA vigorously, gDNA/Cas9 plasmids or gRNA/Cas9 mRNA microinjection usually results in a high success rate of GE pig generation. However, surgical harvesting of fertilized eggs, microinjection, and surgical ET require intensive skills available chiefly in research institutes and thus can hardly be practiced on a farm. Thus, a realistic approach needs to be established for the future extension or commercial uses of GE pigs.

In addition to microinjection, electroporation is advantageous, as it can vastly treat *in vitro*-fertilized zygotes with gRNA and Cas9 protein to produce GE embryos [13], and by applying it, *GGTA1* KO [14] and CD163 KO pigs [15] were successfully generated. However, using oocytes harvested from a slaughterhouse leads to the problem of having an unknown genetic background of the oocytes, and such an approach therefore is absent in the pathways of Figure 1. Instead, to source cells derived from elite stock is the best choice for adding value to existent superior performance stock. Obviously, electroporation is the ultimate process in cell transfection and iPSC/GE induction, especially using plasmid vectors without the integration of any transgenes.

### Induced pluripotent stem cells

GE somatic cells usually need a selection reporter, e.g., fluorescent or antibiotic resistance genes, to assist in edited cell screening [16]. However, when those cells are used as nuclear donors and cloned, the becoming animals will be considered genetic modification organisms (GMOs) and legally prohibited or regulated before entering the food chain. When the GE is simultaneously arranged with iPSCs and prescreened, the definite genotype carried can be confirmed to contain no exogenes. Thus, after iPSC reconstruction, the animals developed are not transgenic and should be exempt from GMO regulations and treated as normal farm animals.

**Figure 2:**
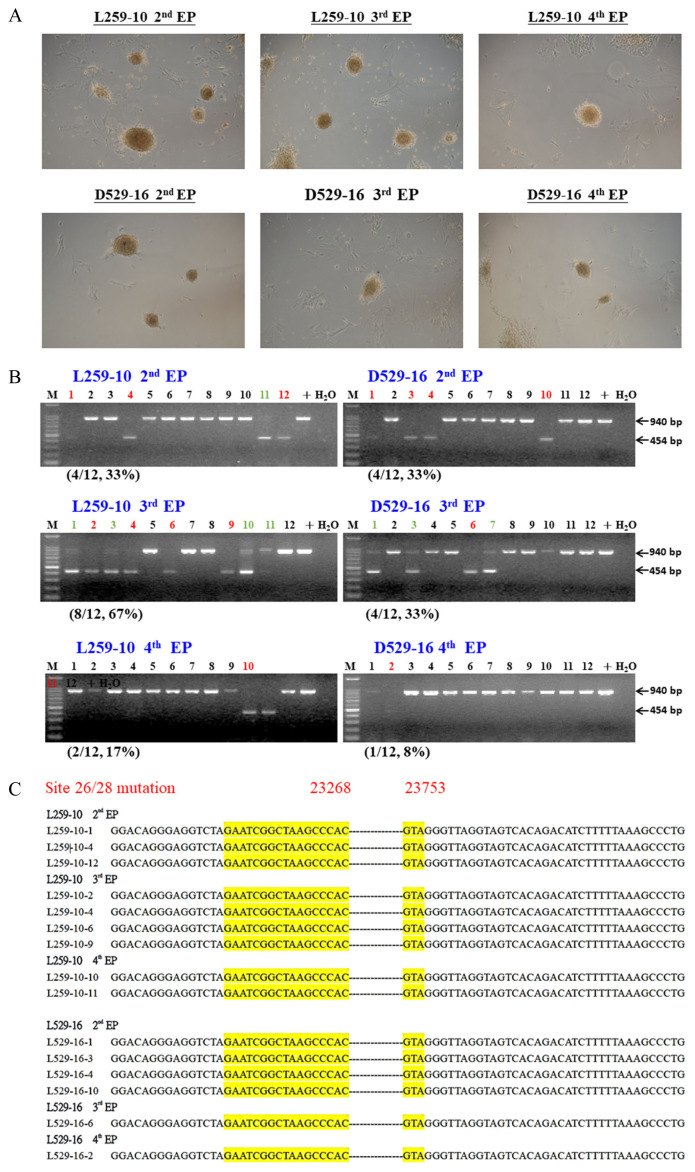
Generation of pig CD163 exon 7-edited porcine induced pluripotent stem cells (piPSCs) by transfection of iPSC-induced factors and CRISPR/Cas9 gene-editing plasmid vectors by electroporation. (A) The morphology of piPSCs after two (2nd EP), three (3rd EP), or four (4th EP) electroporations; the treated primary fibroblast cells were established from a neonatal piglet, L259-10, and an adult sow, D529-16. (B) All candidate piPSCs were analyzed by genomic DNA PCR. The red color is homologous piPSCs carrying double chromosome CD163 exon 7 deleted, and the green colors are heterologous; the numbers and % in parentheses indicate the efficiency of homologous GE. (C) PCR amplicons from all homologous GE/CD163 KO piPSCs were further confirmed by DNA sequencing, and exon 7 of the *CD163* gene was proven to be deleted. CRISPR/Cas9, clustered regularly interspaced short palindromic repeat-CRISPR associated protein 9; PCR, polymerase chain reaction; GE, gene-edited.

In 2006, Takahashi and Yamanaka [17] reported that only four transcription factors, *Oct4*, *Sox2*, *Klf4*, and *c-Myc* (OSKM), are needed for inducing mouse iPSCs. In 2009, porcine iPSCs (piPSCs) were successfully generated by three different research groups [18–20]. Then, in 2010 and 2011, West et al [21,22] produced germline transmitted chimeric pigs from piPSC. Furthermore, Liu et al [23] generated piPSCs by using porcine *Oct4* and *Klf4* combined with small molecules, and their pluripotency was proven by the formation of teratomas. However, since then, no other piPSCs have been reported regarding chimeric pig generation due to exogene issues, even using morulas injected with iPSCs or cloned 4-cell embryo aggregates [24]. To avoid this concern, we established pCX-p*Oct4*-2A-h*KLF4*-2A-p*Sox2* (*pCX-OKS*), *pCX-pcMyc*, and *pCX-hAID-2A-hTDG* plasmid DNAs; by using these plasmids, primary mouse fibroblast cells were successfully induced into iPSCs and characterized by chimeric mouse generation with germline transmission capability [25]. An improved pCX-*pOct4-2A-pSox2-2A-pKlf-2A-hNANOG* (*pCX-OSKN*), *pCX-pcMyc*, and *pCX-TAg* plasmid cocktail was developed and routinely utilized to induce the obtained primary fibroblast cells into piPSCs (Figure 2A) for research in pig production and medical applications.

Currently, human iPSCs have been widely studied; the trends in clinical trials with potentials in several tissues have been recently reviewed [26]. Apparently, before the cells can be clinically used, the genome of iPSCs should be edited and/or modified. In 2015, Howden et al [27] first used patient fibroblasts for simultaneous reprogramming and gene correction to generate DNA methyltransferase 3B (*DNMT3B*) and *Oct4* with green fluorescent protein (GFP) genes in KI iPSCs, but only 3% to 5% of their iPSCs expressed GFP. The same strategy was followed in numerous studies, and we used the same method to generate CD163 exon 7-deleted (CD163ΔE7) piPSCs (Figure 2A). After PCR screening (Figure 2B) and amplicon sequencing (Figure 2C), the candidates could simply be confirmed, with 8% to 67% (Figure 2B) of piPSCs suitable for further cloning uses.

However, difficulties remain in using piPSCs as nuclear donors for SCNT to clone pigs. Du et al [24] used 6 piPSC lines, which all expressed iPSC-inducing genes but failed to generate any cloned pigs; cloning early-stage embryos was also possible by using piPSCs as nuclear donors for SCNT [28–30]. Live piglets were finally successfully generated by silencing exogenous transcription factors and increasing histone acetylation [31] or restoring the expression of the imprinted gene retrotransposon Gag like 1 (*RTL1*) to minimize fetal loss due to post implantation failure [32].

### Somatic cell nuclear transfer

Three years after the success of sheep cloning [33], cloned pigs were generated by three different groups in 2000 [34–36]. The generation of cloned pigs remains problematic and is labor intensive and inefficient; approximately 0.3% to 2% of transferred embryos can develop into live piglets [36]. The challenges are derived from the type/cycle of donor cells selected, methods of oocyte enucleation and nuclear reconstitution, reprogramming/epigenetics of reconstructed embryos, zygotic gene activation, and IVC systems. The added shortcomings inevitably hinder the development and implantation of reconstructed embryos, and the concerning aspects of pig cloning are briefly discussed.

In donor cell selection, fibroblast cells from fetal tissues, ear [37], kidney [38], cumulus cells [39], adipose-derived [40] and bone mesenchymal stem cells [41], and recently extraembryonic endoderm cells [42], have all been successfully used to generate cloned embryos or pigs. We suggest that fibroblasts from neonatal piglets delivered from elite sires and dams with farm-preferred traits are a better source than others for generating GE and cloning pigs to maintain genetic diversity in pig production. The donor cells were usually starved with 0.5% fetus calf serum (FCS) for 3 to 5 days to synchronize the cell cycle at the G0/G1 stage. However, evidence showed that 5 days of starvation of donor cells with no serum improved blastocyst formation rate, but H3K9me3 levels remained unchanged [43], and the cells with size d≤13 μm exhibited a higher percentage at the resting/proliferative (G0/G1) stages with a better proliferation ability [44]. Since chemical-assisted enucleation was firstly developed in conventional SCNT by Yin et al [45], and used in handmade cloning [46], the nocodazole or demecolcine are commonly used for protruding second polar body and followed by sucking out of partial cytoplasm near the polar body of MII oocytes.

The reconstitution approaches include electrofusion of subzonal donor cells with enucleated oocytes [47], direct microinjection of donor cells into the cytoplasm of enucleated oocytes [36], or electrofusion of 2 bisected enucleated oocytes with one donor cell [48]. After reconstitution, the embryos will be further activated by electricity or chemical agents. Recently, a simple and more reliable chemical activation by ionomycin/TPEN (*N*,*N*,*N′*,*N′*-tetrakis[2-pyridinylmethyl]-1,2-ethanediamine) was established [49], and by a similar treatment, cloned pigs were successfully generated [50]. The reconstructed embryos were further *in vitro* cultured in NCSU-37 or PZM-5 medium and then surgically transferred into the recipients on the next day or after the cleavage rate and blastocyst formation rate were measured 2 and 5 to 6 days later, respectively, and surgical ET or NSET [44] to surrogates. However, the development of reconstructed embryos is vulnerable to reprogramming complications [51], including genomic DNA methylation [52], histone deacetylation and methylation [51], apoptosis [53], endoplasmic reticulum oxidative stress [54], heteroplasmic mitochondrial DNA [55], and X-chromosome inactivation [51]. A recent review described in detail the strategies to improve the efficiency of SCNT [56].

The complexity of reprogramming of reconstructed embryos mostly occurs on the methylation of CpG islands of genomic DNA [52], histone deacetylation on histone 3 lysine 9 acetylation (H3K9ac) and H3K14ac, and methylation of histone 3 lysine 4 trimethylation (H3K4me3), H3K9me3 and H3K27me3 [51]. In genomic genes, DNA methyltransferases (DNMT families, including DNMT1 and DNMT3) are involved in the methylation of cytosine (5-methylcytosine, 5mC) on CpG islands [57] in cell differentiation. During oocyte maturation, 5mC can be oxidized by ten-eleven translocation 3 (Tet3) DNA dioxygenase to hydroxylmethylcytosine (5hmC) [58]. With the addition of vitamin C (Vit. C) to IVC medium, the implantation of mouse embryos could be improved, because 5mC is oxidized into 5hmC [59], and also to enhance the SCNT porcine blastocyst development rate [60,61]. In addition, Vit. C was found to remove approximately 40% of the methyl group of genomic DNA by oxidizing 5mC into 5-formylcytosine (5fC) and 5-carboxylcytosine (5caC) by Tet DNA dioxygenase [62]. This vitamin could also reduce the methylation content in SCNT porcine embryos [63] and enhance zygotic genome activation (ZGA) [64]. Evidently, Vit. C is beneficial for porcine reconstructed embryo development and should be added to the medium from IVM to IVC.

The most complicated reprogramming occurs in histone deacetylation and methylation, which influence ZGA. Histone deacetylation can be inhibited by trichostatin A (TSA) [65], which also induces apoptosis in SCNT embryos [66]. Instead, using 500 nM scriptaid (6-[1,3-dioxo-1H,3H-benzo(de)isoquinolin-2-yl]-hexanoic acid hydroxyamide), 80% of the recipients could be impregnated, and farrowing live cloned piglets using scriptaid is a better choice than TSA [67]. Moreover, scriptaid not only increased acetylation levels on H3K9ac but also reduced H3K9me3 and apoptosis, including increasing *Bcl-xl* and decreasing *Bax* and *Casp3* expression [68]. A similar result with several beneficial effects has also been obtained by using chaetocin [69].

In histone (H) methylation, the DNA coiled on the surface of the nucleosome, which is composed of 2 sets of H2a, H2b, H3, and H4 [70], and the methylation occurred at the 4, 9, and 27 lysine (K) of the H3 tail and was represented by H3K4me3, H3K9me3, and H3K27me3 along with the disruption of SCNT embryo reprogramming [51]. By comparing the methylation of IVF and SCNT embryos, the most variance occurred at H3K4me3 rather than at H3K9me3 and H3K27me3 [51]. The intensity of H3K4me3 at 2 to 4 cell embryos from IVF was higher than that from SCNT, but the difference was inverted in 8-cell stage embryos [51]. Recently, lysine demethylase (KDM) has been proven to reduce H3K9me3 content and improve SCNT mouse blastocyst formation [71], and after SCNT, the injection of *Kdm4d* mRNA resulted in a higher number of live-born cloned mice [72]. The benefits of using *KDM4A* mRNA in human blastocyst cloning [73] and *Kdm4d* mRNA in monkey cloning [74] were also found.

In porcine SCNT, Weng et al [75] injected *Kdm4a* mRNA into reconstructed embryos, which resulted in a higher blastocyst formation rate and cell numbers; the same advantage was also gained by adding only chaetocin into IVC medium after activation, as chaetocin, a 3–6 epi-dithio-diketopiperazine secreted by *Chaetomium minutum*, inhibited methyltransferase activity [76]. Recently, by adding chaetocin to IVC medium for 24 h after activation, Jeong et al [77] improved reconstructed embryonic development, including the cleavage rate, blastocyst formation rate and cell number, and increased *Oct4*, *Nanog*, *Sox2*, *Cdx2*, *Bcl2*, and *Bcl-x1* gene expression, but *SUV39h1* and *SUV39h2* (DNA methyltransferase genes) expression and H3K9me3 content were decreased. By supplying chaetocin and TSA at the same time, the reconstructed embryo development and the levels of H3K9me3 and H3K9ac in SCNT embryos were further improved; moreover, the expression of ZGA- and imprinting-related genes was also increased [78].

The SCNT comprises enucleation, donor cell injection, and *KDM* mRNA injection; those labor-intensive micromanipulation are inefficiency with low success rates and thus discouraging GE pig generation for potential farm uses. Improving the efficacy of chemical enucleation is vital for oocysts; direct cytoplasm injection with donor cells to minimize abnormal reprogramming by using IVC with TSA/scriptaid/chaetocin instead of *KDM* mRNA should be the best option.

### Vitrification of porcine cloning embryos

In 1993, Yoshino et al [79] developed a 4-step vitrification procedure by using a high concentration of cryoprotectant to preserve porcine expanding blastocysts, making them remain transparent (no ice crystals) under a fast cooling rate without a machine freezer. Katayama et al [80] cryopreserved human oocytes by adding 7.5% ethylene glycol (EG)/7.5% dimethyl sulfoxide (DMSO) and 15% EG/15% DMSO/0.5 M sucrose (S) into basal medium (BM; containing 20% FCS) as equilibration solution (ES) and vitrification solution (VS), respectively. After thawing, IVF and other procedures, pregnancy was achieved. The protocol was successfully applied in the cryopreservation of delipidate IVC porcine embryos [81]. Cuello et al [82] used 17% EG/17% DMSO/0.4 M S VS and achieved an 89.2% to 95.5% survival rate in expanded porcine blastocysts after thawing. In 2008, they further compared different concentrations of VS with 40% EG with 15% EG/15% DMSO/0.4 M S, 16% EG/16% DMSO/0.4 M S, or 17% EG/17% DMSO/0.4 M S; and obtained a similar survival rate between different VSs [83].

Using vitrified SCNT embryos, live piglets were successfully generated by cloned and delipidated blastocysts [84] or morulas [85], which were cryopreserved with the same protocol of EG/15% DMSO/0.5 M S VS. Recently, Jia et al [86] used 15% EG for ES and 50 mg/mL PVP/35% EG/0.6 M S of VS to cryopreserve the zygotes, 2 and 4 cells of constructed porcine embryos on Crotop carrier, and achieved a 97.3% to 98.1% survival rate and 21.1% to 35.8% developing rates of BC after thawing. The advance of porcine embryo vitrification has been well reviewed by Du et al [87] recently, and further searching for better cryoprotectants, e.g., carboxylated epsilon-poly-L-lysine [88], together with endeavors to increase the viability of SCNT embryos would certainly facilitate the application of GE in pig breeding.

### Nonsurgical porcine embryo transfer

The NSET of BC or morula, but not 8-cell or 4-cell porcine embryos, when transferred to sedated sows resulted in live piglets born as first reported by Day’s research team in 1996 [89]. The practice was subsequently used on sober sows, and farrowing rates of 17% and 41% were obtained when *in vitro* [90] and *in vivo* [91] BC were used, respectively. By using NSET, Cuello et al [92] transferred the vitrified BC into gilts, with 2 to 6 previous estrous cycles, at 5.5 to 6 days after showing standing heat and found that the flexible catheter could not pass through the cervix in all the gilts with 2 previous cycles. In the gilts with 3 to 6 previous cycles, 80.9% reached the second or third quarter of the uterine horn, and among the gilts, 42.9% became pregnant and farrowed 3 to 9 piglets. Other attributes of a successful NSET include a suitable transferring volume of 1 to 2 mL medium [93]; dish warming of vitrified embryos performed better than syringe thawing [94], and a catheter should be inserted into the uterine horn at least 30 cm deep [93]. The recipient’s parity, when varied from 1 to 5, had little effect on the piglet production efficiency [95].

With respect to the farrowing rate, the synchronization of recipients with a day delay (81.1%) was better than a day early (0%), synchronous (61.3%) or 2-day delay (50%) if fresh embryo donors were transferred [96]. While transferring the vitrified/warmed expanded BC, 2-day and 1-day delays gave 27.3% and 25.0%, respectively [97]. Recently, a simplified NSET procedure was suggested by transferring the vitrified BC at the proximal site of the uterus (uterus body) to minimize the needs of experience and skill, but the farrowing rate was only 15.4% [98]. A more satisfactory result of a 42.9% farrowing rate and 6.4% piglet survival rate was obtained on a farm using the same approach when NSET vitrified BC and expended BC [99], and AI before NEST (on 4 farms) increased the pregnancy rate and delivered live piglets from vitrified/thawed embryos [96]. NSET has now become an essential reproductive biotechnology for improving the pregnancy/farrowing rate.

## CONCLUSION AND PROSPECTIVE

Successful farm-extending NBT depends on the synergistic effect of GE and reproductive biotechnologies and is currently ineffectual and has complications. The obstacles coupled with GE/iPSC, SCNT/IVC, cryopreservation, and NEST intensify existing vulnerabilities and magnify loss. Overcoming hurdles across piPSC-SCNT that involve the reprogramming of reconstructed embryos is currently the main concern, and improving the protocol of embryo cryopreservation/banking follows. To facilitate GE pig generation, efforts should not only be exerted on scientific due diligence and operational excellence but also on the feasibility of application on farms.

In the wake of the food crisis fueled by climate change, GE food animals with sustainable traits will soon be considered non-GMOs and will be commercially used. Over the next decade, a significant number of GE pigs produced with economic, environmental, and social values are foreseeable for the marking of the fourth revolution of animal agriculture. Recent advances in biotechnology are offering techniques that optimize the efficiency of GE animal production; therefore, a substantially higher chance of a satisfactory outcome can be anticipated.
